# Impact of cybersecurity measures on improving institutional governance and digitalization for sustainable healthcare

**DOI:** 10.1371/journal.pone.0274550

**Published:** 2022-11-15

**Authors:** Hafiz Syed Mohsin Abbas, Zahid Hussain Qaisar, Ghulam Ali, Fahad Alturise, Tamim Alkhalifah

**Affiliations:** 1 College of Public Administration, Huazhong University of Science and Technology, Wuhan, P.R. China; 2 Department of Computer Science, NFC Institute of Engineering and Technology, Multan, Pakistan; 3 Department of CS, University of Okara, Renala Khurd, Pakistan; 4 Department of Computer, College of Science and Arts in Ar Rass, Qassim University, Ar Rass, Qassim, Saudi Arabia; LUMSA: Libera Universita Maria Santissima Assunta, ITALY

## Abstract

Digitalization in healthcare through advanced methods, tools, and the Internet are prominent social development factors. However, hackers and malpractices through cybercrimes made this digitalization worrisome for policymakers. In this study, the role of E-Government Development as a proxy for digitalization and corruption prevalence has been analyzed in Healthcare sustainability in developing and underdeveloped countries of Asia from 2015 to 2021. Moreover, a moderator role of Cybersecurity measures has also been estimated on EGDI, CRP, and HS through the two-step system GMM estimation. The results show that EGDI and CRP control measures significantly improved HS in Asia. Furthermore, by deploying strong and effective Cybersecurity measures, Asia’s digitalization and institutional practices are considerably enhanced, which also has an incremental impact on HS and ethical values. This present study added a novel contribution to existing digitalization and public health services literature and empirical analysis by comprehensively applying advanced econometric estimation. The study concludes that cybersecurity measures significantly improved healthcare digitalization and controlled the institutional malfunctioning in Asia. This study gives insight into how cybersecurity measures enhance the service quality and promote institutional quality of the health sector in Asia, which will help draft sustainable policy decisions and ethical values in the coming years.

## 1. Introduction

One of the key pillars of the United Nations’ Sustainable Development Goals (SDGs) is healthcare sustainability (HS) [1,2]. Leading international organizations, like the World Bank, WHO, UN, and Asian Development Bank, assist and invest in different country segments to improve their institutional capacity and quality to achieve the goal. Governments have been working to improve their public services and make them more usable by collaborating with regional partners and state resources. Information and communication technology (ICT) can improve the accessibility and effectiveness of public services [3–8].

The current hot topic in improving sustainable public services is ICT inclusion and related investment. Since the SDGs were announced, every country has arranged IT deployment services, regardless of its level of development. According to research, wealthy nations are making significant expenditures on their electronic governance systems. But important components of this deployment are state capacity and ICT development. These factors are not met by developing and growing nations and lack an efficient electronic governance framework [9–13].

Despite several e-government projects and activities, some prevent efficient e-government system implementations [14]. The UN has also concentrated on making the e-government development initiatives secure and error-free since the launch of the SDGs by creating the Cybersecurity index through its Information Technology Unit. The UN’s ITU creates awareness and organizes numerous activities to strengthen secure and sustainable e-government systems [15]. The risk of cybercrimes and hacking increases as the governance and service systems become more digital, which is a significant barrier to inefficient electronic service delivery [16]. A stronger cybersecurity system will protect organizational, practical, legal, and public databases and increase public trust in the government through development [17–19].

Corruption is a significant concern for adequate institutional quality, along with cybersecurity, a problem that affects a large portion of society. Transparent or anti-corruption services are required for better institutional governance and policy implementation. The Transparency International [20] survey indicates that public institutions and employees are more vulnerable to corruption. According to a recent study on the relationship between corruption and public services, corruption impacts public services in Asian economies [21].

The economy may be impacted by malware and Trojans that pose legitimate technical services applications. Banking Trojans can interfere with legitimate banking transactions. Trojans and ransomware can occasionally result in the theft of money and important credentials. Cybercriminals can target banking systems, interfere with the seamless completion of normal tasks, and further their own economic, political, and hidden social agendas [22,23]. Weems et al. [24] used a computing-based decision-making system to improve the E-government program and manage cyber threats; however, because their studies were conducted in European nations, there is still an opportunity for further research in other regions.

This study was created to assess the relationship between digitalization and security by using the proxy of e-government development with HS because of the significance of e-government and security. Additionally, a brand-new moderation analysis of cybersecurity has been used. This moderation analysis aims to assess how cybersecurity measures affect the states of UN members and how they affect digitalization and institutional practices for long-term public services. Asia’s developed and underdeveloped economies have been chosen as a study sample. Studies have been done on the effects of e-government on specific Asian economies. The researchers were inspired to carry out this study because there aren’t many panel Asian studies in this area, and e-government and cybersecurity regulations are far more restricted than they are for the former. According to the International Monetary Fund-IMF [25], investing in Asia can benefit and significantly impact the global economy. A recent study on a panel of Asian economies was carried out by Abbas et al. (2021). They did include established Asian economies in their sample, however. Because wealthy nations have more advanced cybersecurity and digital infrastructure than underdeveloped nations, our study conducted an accurate analysis by eliminating them.

By incorporating sustainable development literature into the research directions of Baker [26], Adjei-Bamfo et al. [27], Ulla et al. [7], Shah et al. [28], and Abbas et al. [21]., the current study contributed value to e-government and the role of ICT. Utilizing the right ICT services and innovations to examine the obstacles to its execution will improve e-government practices. By adopting an e-government system, reducing cybersecurity threats, and policing institutional malpractices, the findings will assist in developing good and sustainable health services policies. This study can assist policymakers in drafting policies considering the trends of e-government development, cybersecurity, and sustainable public services by considering the relationship between ICT with related risks and public services.

## 2. Literature review and hypotheses development

### 2.1 Sustainability in healthcare

The worldwide projection of national and regional development is known as sustainable development. The United Nations, the World Bank, and the United Nations Development Program are just a few of the world’s top organizations paying close attention to the SDGs and making wise policy decisions in this area (UNDP). Various internal and external factors impact sustainable development in developing and growing economies [29,30]. Quality and accessibility of public services are essential to achieving the SDGs, particularly in rising and developing economies. In southern Asian countries, Larsson [31] examined state governance and policymaking as important institutional performance and service delivery determinants. Cohodes [32] explored how a state’s capacity and wise resource management can close the development gap and ensure long-term health.

The impact of state policy, accountability, and capability on Australia’s public services was examined by Ferguson [33]. Even in wealthy states, he examined how innovation in state resources and good policymaking affected the quality and delivery of public services. Institutional quality and policy choices have a significant impact on the quality and delivery of healthcare, according to Ramasamy’s [34] study in Sri Lanka. Using GMM estimates, Abbas et al. [21] investigated the influence of institutional governance and state fragility determinants on public service quality in Asia. They found that efficient institutional innovation, the use of human capital, and resource management improve service delivery and quality while lowering the fragility of SD’s service.

### 2.2 E-government development and sustainability in healthcare

A thorough e-government development index combines online services, human capital usage, and telecom services [13,35,36]. Simply put, e-government is using ICT to improve the delivery of governmental services. A robust network of communication between the government and the people is created by an efficient and reliable e-government system, which also improves service delivery. A safe and cutting-edge ICT system makes health services more convenient and accessible in this age of digitization by protecting public data and e-health services. In China, the effects of ICT or e-government systems on institutional quality and service were studied by Guanghua [37], Schlaeger [38], and Hung [39]. They looked at how digital transformation programs have boosted productivity, human capital capabilities, and public services in public organizations. It also investigated how implementing safe and reliable digital transformation platforms enhanced service delivery and public confidence [40–43].

Despite global issues and well-being, the UN’s SDGs emphasize developing and emerging economies and their improvement [36]. In emerging African and Latin American states, digitalization is increasingly impacting the effectiveness and quality of services [5,6,8,44]. They talked about how these emerging regions have advanced over time and enhanced service sustainability thanks to online service delivery and human talents. Service delivery is now quick, effective, secure, and inventive, thanks to digitization. They contend that the digital transition has improved living conditions, health care, and educational standards in developing nations considerably more than in recent years.

During the COVID-19 epidemic, there was a clear need for ICT performance and efficiency, and China has excelled in this area [45]. Furthermore, developing and emerging nations have combatted the pandemic despite having fewer resources. To address COVID-19, Ceesay, Bojang, [9] and Iyamu [10] looked into how well the health care system performed and how ICT was used to interact with the public in African states. They discovered that it was both satisfactory and improving. In comparing e-government and public policy in China and Pakistan, Ullah et al. [7] discovered that the e-government system considerably impacted institutional quality and performance. In a different study, Abbas, Xu, and Sun [3] systematically analyzed the effects of 5G and 6G advanced digitalization on the Chinese health system and service delivery. They concluded that using technology effectively during COVID-19 in China had given the country an advantage over the rest of the world in its fight against the epidemic. Shah et al. [28] evaluated the private ICT invasion and online public services in the context of e-government in China and Pakistan. They discovered that ICT improved quality of life, helped people save time, and improved accessibility of public services to move toward SD.

### 2.3 Sustainability, corruption, and digitalization in healthcare

Corruption is the biggest societal ill that hinders governmental development and openness [46]. Regardless of the state’s progress and advancement, corrupt institutional practices impair institutional quality and integrity in the same way that pollution degrades the quality of the environment and human life. Moschovis [47] investigated the effects of corruption on public financial management in industrialized and developing European countries. He claimed that until a superb transparency network was implemented in governmental systems, public institutions’ corruption negatively impacted funding distribution and projects. One core tenet of the UN’s SDGs charter and cutting-edge e-government system is eliminating corruption due to its pervasiveness and consequences on institutional quality [35,36,48].

According to Forson et al. [49] and Thompson [50], institutional corruption can be decreased through the theory of corruption by adopting a transparent system for SD and grooming staff individually. Using fixed effect estimates, subsequent research of low- and middle-income nations found that institutional and policy reforms lower corruption [51]. The effects of decision support systems in Brazilian public institutions and corruption control software in Indian institutions on reducing corruption were studied by Velasco et al. [52] and Morya and Singh [53]. They found it appealing and long-lasting to improve institutional performance and openness. In addition, Hooda and Singla [54] and Marchini, Mazza, and Medioli [55] noted that ICT integration reduces the negative impacts of corruption and improves the integrity and transparency of the public sector.

### 2.4 Cybersecurity, digitalization, and sustainable healthcare

The improvement of the e-government system and innovation in service delivery mechanisms depend heavily on the cybersecurity network and system. ICT has revolutionized governance and significantly enhanced the delivery of contemporary services in developing nations [56]. According to others, a reliable cybersecurity system boosts digitization, opens the national network to other countries, and instills public confidence in Asian economies [16,17,57]. According to Andoh-Baidoo, Osatuyi, and Kunene [58], a well-designed cybersecurity framework could improve the quality of cyber systems and service delivery in the sub-Saharan African region despite limited resources. Malhotra, Bhargava, and Dave [59] investigated the concerns about e-government in the Indian and African economies and discovered several dangers and constraints in emerging nations that are barriers to expanding freely available digitalization public services.

Weems et al. [24] used a computer program to control cyber threats using a scenario-based decision-making method. In further research, Prislan, Miheli, and Bernik [60] used the information and security strategy used by the European state to analyze the multidimensional socio-technical approach. They stated that cybersecurity threats might be successfully countered by considering socio-economic variables. Researchers have examined security threats to public institutions and their risk management practices in developed and emerging Asian and European countries by focusing on the SDGs and secure ICT networks [54,61]. They examined how cybersecurity safeguards and ICT standards benefit established and emerging economies’ e-government systems. However, developing nations are significantly more concerned about cyber threats and malfunctions because of their lack of resources and knowledge. Morya and Singh [53], Gupta, Pal, and Muttoo [62], among others, have concentrated on how secure networks can improve the e-government system, increase the state’s ability to address ICT flaws, hasten the sustainability of public services at the national, regional, and international levels, and ensure sustainable digitalization. Adjei-Bamfo et al. [27] conducted primary research to gather information by analyzing the function of e-government evaluation and public sector quality in Ghana and concluding that it was practical. Rothe [63], Shah et al. [28], and Creese et al. [18] investigated the use of e-government and ICT in the provision of public services in south and east Asian nations. They discovered that by carefully considering cybersecurity precautions, which promote sustainable development, ICT has brought value to transportation and health services. Using Fixed Effect and Random Forest algorithms, Abbas et al. [21]. investigated how cybersecurity measures enhanced public services in Asian economies from 2015 to 2019.

## 3. Material and methodology

Public services and digitalization have become popular trends in developing nations. The current study has looked at how digitization affects the viability of health services in developing and underdeveloped Asian nations (see Appendix A). To achieve the goal, a dependent variable of public health services has been used as a stand-in for the sustainability of healthcare [21,64]. At the same time, the corruption prevalence index and the E-Government Development Index have been treated as independent variables. Moreover, the population increase is regarded as a control variable in this analysis, and the cybersecurity index has been chosen as a moderator influencing the effectiveness of the e-government system [65]. See Table 1 for more information on variables and descriptions. The UN has released the data for the cybersecurity index.

**Table 1:** Variables measurement, period, and description.

Variables	Capacity	Time-Frame	Description and Source
Public Services Index, a proxy of Public Health Sustainability (HS)	Dependent Variable	2015–2021	Composite Index of Health Quality, infrastructure, facilities: Fund For Peace
E-Government Development Index (EGDI), a proxy for digitalization*	Independent Variable	2015–2021	Digitalization of Public Services systems via online, human development, and telecommunications networks. Source: The United Nations (UN)
Cybersecurity Index (CYSI)*	Moderating Variable	2015–2021	Government measures to control cyber crimes and secure the e-government. Source: Information Technology Unit, the United Nations (ITU, UN)
Corruption Prevalence Index (CRP)	Independent Variable	2015–2021	Corruption prevalence rate or corruption-free government system. Transparency International (TI)
Population Growth Rate (POP.G)	Control Variable	2015–2021	Population growth rate per anum.Source: World Development Indicators by the World Bank

* For analysis, two years average method has been applied to cover the missing or unavailable data for smooth empirical estimation.

### 3.1 Research framework

**Fig 1:**
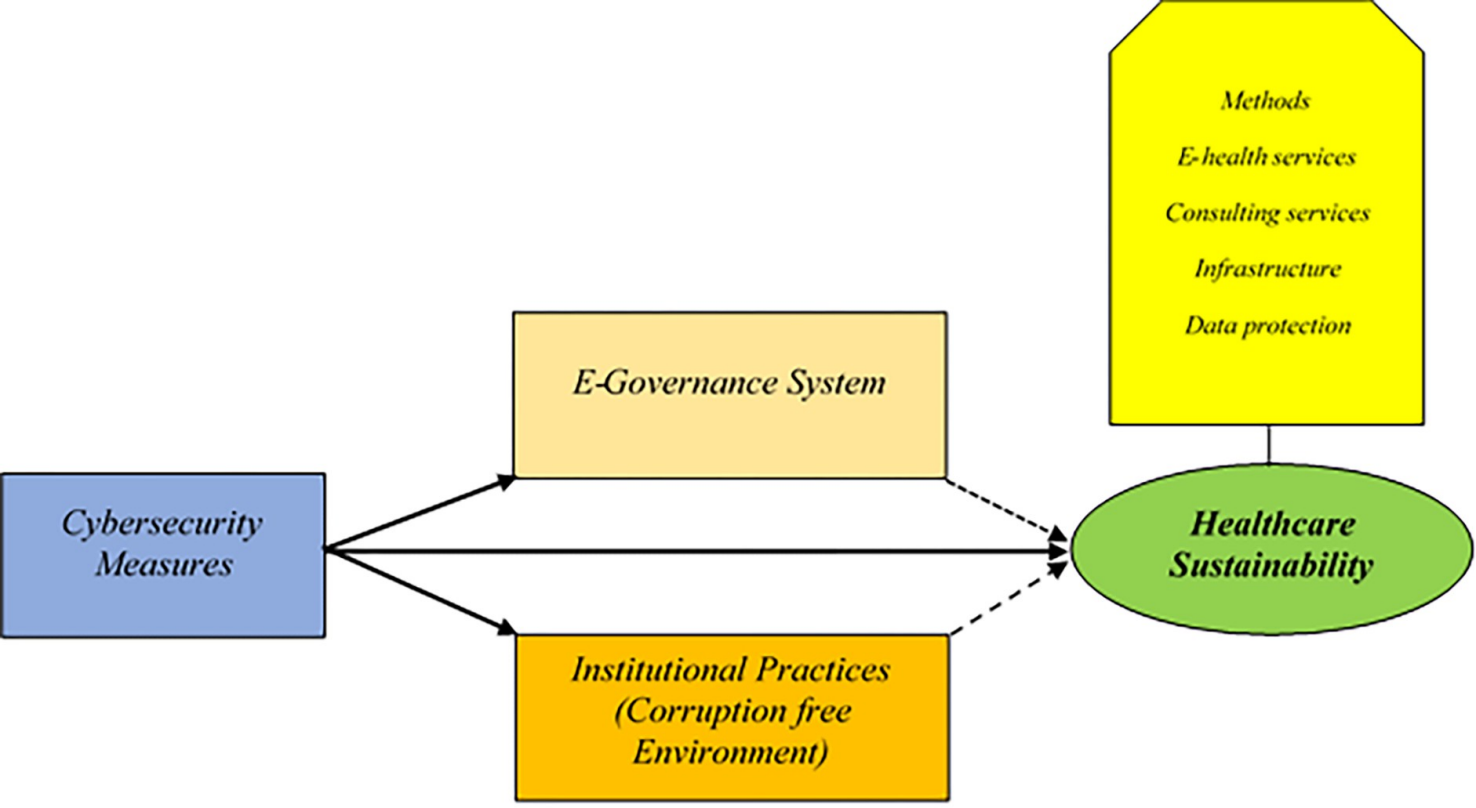
Conceptual framework based on study literature and designed.

Based on the relevant literature and study design, the present study developed a conceptualized research framework in Fig 1. According to the research framework, the e-governance system and corruption-free institutional practices directly impact healthcare quality and are denoted with dotted lines. At the same time, cybersecurity measures with bold lines show a moderator role which improves the institutional controlling or removing the malfunctioning and enhances the digitalization capacity of the institutions that provide health services and achieve sustainability in line with SDGs.

### 3.2 Data analysis strategies

The two step-system Generalized methods of moments is a baseline econometric technique applied in this study. The GMM is an advance used for cross-sectional panel data analysis in Asian economies to investigate the sustainable development nexus with political, financial, and systemic determinants in various studies. The advantage of using GMM in the social sciences field is that it helps control the endogeneity, heteroskedasticity, and data error in the same analysis, especially in panel data analysis. Moreover, it helps in the exact and accurate model estimation [21–23,66–78].

The following econometric model is used in this study based on applied techniques and study design.


HS=∫(EGDI,CYSI,CRP,POP.G) (1)

Direct Channel:

$$HS=\beta_{0}+\beta 1\;(EGDI)i,\tau +\beta 2\;(CYSI)i,\tau +\beta 3\;(CRP)+\beta 4\;(POP.G)\mu_{i,\tau }$$ (2)

Indirect Channel:

$$HS=\beta_{0}+\beta 1\;(HS)i-1,\tau +\beta 2\;(EGDI)i,\tau +\beta 3\;(CYSI)+\beta 4\;(CRP)\mu_{i,\tau }\;\beta 4\;(POP.G)\mu_{i,\tau }$$ (3)

In above Eqs 1–3, HS = Healthcare Sustainability; EGDI = E-government Development Index; CYSI = Cybersecurity Index; CRP = Corruption Prevalence Index; POP.G = Population Growth rate; μ = Error Term; i = Country and τ = Time (year)

## 4. Results and data analysis

### 4.1 Descriptive and correlation statistics

Table 2 describes the descriptive statistics of the study sample from 2015–2021 of 42 Asian economies.

**Table 2:** Descriptive statistics.

Variables	Mean	Std. Dev.	Min	Max	Observations
HS	5.418	2.115	1.300	9.950	294
EGDI	0.552	0.166	0.211	0.856	294
CRP	3.583	1.363	1.100	7.100	291
CYSI	0.470	0.296	0.000	0.995	293
POP.G	1.509	1.131	-3.887	5.791	294

Source: Authors Estimation.

The results of Pearson’s correlation analysis are demonstrated in Table 3. As per statistics, a dependent variable, Healthcare Sustainability (HS), has an inverse relationship with the explanatory variable E-government Development Index (EGDI) and Corruption Prevalence Index (CRP). Moreover, HS also has an inverse relationship with moderating variable Cybersecurity Index (CYSI). All values have a 99% confidence interval.

**Table 3:** Pearson’s correlation analysis.

Variables	HS	EGDI	CRP	CYSI	POP.G
HS	1.000				
EGDI	-0.819***	1.000			
CRP	-0.654***	0.563***	1.000		
CYSI	-0.527***	0.731***	0.444***	1.000	
POP.G	0.053	-0.073*	0.014	-0.218**	1.000

*** p<0.01

** p<0.05

* p<0.1.

Source: Authors Estimation.

### 4.2 GMM A baseline analysis

Table 4 explains the GMM estimation of the study variables. As per statistics, EGDI and CRP have a significant impact with a 99% confidence interval on HS in Asia. In contrast, EGD has a high magnitude impact than CRP. Adding the moderator of CYSI in the analysis shows that CYSI has a significant positive effect on HS. It also indicates that CYSI improved the EGD performance and strengthened the corruption-free environment by providing strong and effective cybersecurity measures in controlling malpractices. It enhances the data management system, online services, and strong and secure internet services to provide efficient healthcare services. All the variables are significant with a 99% confidence interval which support the study model and analysis.

**Table 4:** Two step system GMM Results without and with a moderator.

	*DIRECT*	*With Moderator*
VARIABLES	*Two Step System GMM*	*Two Step System GMM*
HS	*0*.376***	*0*.356***
	*(0*.*0785)*	*(0*.*0829)*
EGDI	*-2*.671***	*-4*.156***
	*(0*.*776)*	*(0*.*937)*
CRP	*-0*.898***	*-0*.931***
	*(0*.*205)*	*(0*.*183)*
POP.G	*-0*.180**	*0*.*0760*
	*(0*.*0795)*	*(0*.*0840)*
**CYSI (Moderator)**		*-1*.069***
		*(0*.*336)*
Constant	*8*.393***	*8*.752***
	*(1*.*099)*	*(1*.*132)*
Observations	*251*	*251*
Number of Countries	*42*	*42*

Source: Authors Estimation.

Table 5 denotes the robust analysis which backed the study results in Table 4. According to the robust estimation, all the tests support the Table 4 results and show the strong relationships of study variables.

**Table 5:** Robust analysis of GMM.

Analysis	*Direct*	*With Moderator*
**AR-1**	*-1*.*26*	*-1*.*250*
**AR-1(p)**	*0*.*007*	*0*.*002*
**AR-2**	*-0*.*650*	*-0*.*820*
**AR-2(p)**	*0*.*200*	*0*.*414*
**Hansen**	*35*.*08*	*36*.*80*
**Hansen (p)**	*0*.*001*	*0*.*003*
**J-Statistics**	*17*	*22*
**Wald Chi2**	*822*.*67*	*2546*.*15*
**Prob. Chi2**	*0*.*000*	*0*.*000*

Source: Authors Estimation.

## 5. Discussion

The two main themes for regional growth are digitalization and sustainable development. Filgueiras, Cireno, and Palotti [44], as well as Baker [26], Furuholt and Saeb [5], Verkijika and De Wet [8], Rothe [63], and others, have stated that the e-government systems in African nations and latent America are effective in enhancing public services. Since most countries in that region are low-income or developing, there are obstacles related to limited resources and the adoption of cutting-edge technologies that slow progress. The current study showed that e-government development had advanced considerably more quickly than in Asian economies since most nations have a mix of emerging and integrating economies, and established countries also have a big impact on regional development. This study has identified patterns in Asia’s public service fragility and the nexus between e-government. The graphical illustration and empirical findings demonstrate how e-government has greatly lowered the fragility of the public sector and enhanced service quality and sustainability across Asia.

This study with e-government development to public services has studied a novel moderation panel influence on cybersecurity quality and measurements. Researchers have researched the impact of cybersecurity measures aimed at a sustainable and safe e-government system on institutional growth and public service quality in African and single Asian economies using various econometrics and machine learning methodologies [18,22,53,62]. They emphasized how developing African nations struggle to combat cybercrime due to inadequate state capacity and competence. However, since the release of the SDGs, growing Asian nations like China and India have enhanced their cybersecurity measures and played a significant role in the growth of e-government.

Shah et al. [28] and Meland, Bayoumy, and Sindre [23] also made the case that cybersecurity measures weaken cybercriminal networks and give the public legitimacy necessary for digitalization to be secure and effective. The current study tapped into a regional panel examination of how cybersecurity affects e-government and public service quality in Asian nations. According to statistics, enhanced and secure cybersecurity measures have supported and enhanced public services and sustainable development while promoting e-government and ICT throughout Asia.

This study provides an example of how corruption may moderate the growth of e-government in the public sector. The threat of corruption to state progress and development is a global problem [47,48]. It looked at how ICT integration decreases and manages corruption in underdeveloped nations [54,55].

There is a wealth of literature on institutional corruption in Asia, Africa, and other developing and developed regions. In most Asian economies, the impact of corruption in the e-government system on public service is still unstudied. This study used empirical analysis to show how the use of ICT for corruption control positively impacted the quality and fragility of the public service [21]. However, the study shows that because some of the activities of corruption control overlap with cybersecurity networks, they have a smaller impact on the moderation of e-government systems in Asia than secure cybersecurity measures.

## 6. Conclusion and policy implications

Since the ITU and SDGs established their targets of SD by 2030, the study sought to examine the influence of digitalization on healthcare sustainability with the moderation of cybersecurity measures in 42 underdeveloped and developing Asian economies from 2015 to 2021. Public health services have been used as a stand-in for sustainable healthcare, with the e-government development index and an environment devoid of corruption serving as the independent variables and the cybersecurity index serving as the moderator. The two stem system GMM estimate has been used for empirical and computational assessments.

This study significantly contributed to the empirical analysis and literature on digitalization and public health care by thoroughly utilizing modern econometric estimation. It was discovered that e-government advancement had boosted Asia’s accessibility and delivery of public services.

### Future research

In future studies, this model can be applied to other sustainable development goals such as economic growth, energy resources, and public offices working by taking other proxies. This study model can be implemented in further regional analyses predominantly in African countries and a comparative analysis of Asia, Africa, and other developed regions as well, which would be an excellent study direction.

## Supporting information

S1 Dataset (XLSX)
